# Negative and positive emotional complexity in the autobiographical representations of sexual trauma survivors

**DOI:** 10.1016/j.brat.2020.103551

**Published:** 2020-03

**Authors:** Georgina Clifford, Caitlin Hitchcock, Tim Dalgleish

**Affiliations:** a Medical Research Council Cognition and Brain Sciences Unit, University of Cambridge, 15 Chaucer Road, Cambridge, CB2 7EF, UK; b Cambridgeshire and Peterborough NHS Foundation Trust, UK

**Keywords:** Emotional diversity, Emotion complexity, Emodiversity, PTSD, Depression, Self-concept, Autobiographical memory, Sexual trauma

## Abstract

This study examined the diversity of experienced positive and negative emotions – emodiversity – within two existing datasets involving female survivors of sexual abuse and assault, who all met criteria for chronic Posttraumatic Stress Disorder (PTSD) as well as a diversity of comorbid diagnoses. Study 1 investigated the structure of the self-concept and Study 2 explored the organization of past autobiographical knowledge. In each study, we measured emodiversity for positive and negative emotion constructs in the trauma sample, relative to healthy control participants with no history of sexual trauma or PTSD. Results confirmed our hypotheses that individuals with a severe sexual trauma history and resultant PTSD would show elevated negative emodiversity and reduced positive diversity across both the structure of the self-concept and the structure of the life narrative, relative to control participants. The current results differ from community studies where greater negative emodiversity is associated with better mental health but mirror those from a prior study with individuals with Major Depressive Disorder. This suggests that valence-based differences in emodiversity may result from chronic emotional disturbance.

## Highlights

• Examined diversity of experienced positive and negative emotion in trauma survivors.
• Emotional diversity was measured for self-concept and the autobiographical past.
• Trauma was related to increased diversity for negative emotion, relative to controls.
• Trauma survivors also showed reduced diversity for positive emotion.

## 1 Introduction

Emotions are fundamental to human experience. Historically, research indicates that high levels of positive emotion and low levels of negative emotion are an essential component of good mental health and subjective well-being (e.g., Fredrickson, 2001). However, there is increasing interest in how the complexity of emotion experience, over and above this simple balance of positive and negative felt emotions, can underpin mental health (Feldman-Barrett, 2017). The richness and complexity in people's self-reported experience of emotion is a primary aspect of the broad concept of *emotional complexity* (e.g., Lindquist & Barrett, 2008), which has been linked to adaptive emotion regulation and mental health (Labouvie-Vief & Medler, 2002; Ryan & Deci, 2001).

Emotional complexity has been operationalized in a variety of ways, which can be grouped into two broad categories: emotional granularity and emotional covariation. Granularity (Feldman-Barrett, 1998, Feldman-Barrett, 2004; Feldman-Barrett, Gross, Christensen, & Benvenuto, 2001) refers to the degree to which individuals can describe their emotional life with precision, using discrete emotion descriptors to precisely characterize different emotions. Covariation, or dialecticism, refers to the experience of both positive and negative emotional states in a contemporaneous way, or in ways that are temporally related (Bagozzi, Wong, & Yi, 1999). Emotional granularity and emotional covariation measures have been reported to capture important aspects of the complexity of one's emotional life (Quoidbach et al., 2014). For example, the propensity to experience positive and negative affect independently (‘granularity’) has been linked to various indicators of adjustment (Carstensen, Pasupathi, Mayr, & Nesselroade, 2000; Coifman, Bonanno, & Rafaeli, 2007; Reich, Zautra, & Davis, 2003).

Recent research has focused on the *diversity* of emotion experience that an individual reports, and questions have been asked regarding whether the *diversity* within an individual's emotional life is beneficial for greater mental and physical health (Quoidbach et al., 2014; although see Sommers, 1981, for discussion of ‘emotional range’). The thesis is that there are individual differences in how people understand, interpret and communicate the range and nature of their own emotion experience. Some people are more aware of and able to articulate a wide range of discrete, specific emotions while others are more prone to represent how they feel with a relatively narrow set of specific descriptors alongside more global self-characterizations of their emotional life (e.g., feeling ‘good’ or ‘bad’). It has been suggested that a wider range of experience of specific, differentiated emotional states (e.g., happiness, excitement, and exhilaration) has greater adaptive value than a narrower range and than less differentiated, more global affective states (e.g., feeling good). The theory is that differentiated emotional states can be more easily identified and understood by others around us, and are less subject to misattribution (e.g., Kehner, Locke, & Aurain, 1993). Moreover, differentiated emotional states are also argued to provide richer information to guide the use of specific strategies to effectively regulate emotion (Feldman-Barrett et al., 2001; Feldman-Barrett & Gross, 2001). This approach fits within a broader view that biological and psychological flexibility is beneficial for adaptive mental functioning and promotes greater resistance to disease (e.g., Kashdan & Rottenberg, 2010).

Quoidbach et al. (2014) examined the psychological and mental health benefits of such greater emotional diversity – or *emodiversity* as it has been termed. The concept of emodiversity was derived from the literature on biodiversity (i.e., the variety and differential prevalence of different types of life forms within an ecosystem). Greater biodiversity has been associated with adaptive flexibility and greater resilience within an ecosystem (Danovaro et al., 2008; Elmqvist et al., 2003; Heller & Zavaleta, 2009; Potvin & Gotelli, 2008; Rammel & van den Bergh, 2003; Tilman, Reich, & Knops, 2006). Quoidbach et al. (2014) therefore adapted the Shannon Biodiversity Index (Shannon, 1948) to quantify emodiversity using two domains: the richness (how many specific emotions are experienced); and evenness (the extent to which specific emotions are experienced in the same proportion) in what they described as the *‘human emotional ecosystem.’* Their hypothesis was that greater emodiversity would be associated with comparable benefits in emotional and physical health, over and above the frequency of positive and negative emotions experienced.

Quoidbach et al. (2014) surveyed 37,000 participants in the general population to measure symptoms of depression and the self-reported diversity of experienced emotion (emodiversity). They computed three emodiversity indices (positive, negative, and global) using the formula derived from the Shannon Biodiversity Index. The richness and evenness of an individual's emotional experience was computed for each of the three indices. Results suggested that greater levels of emodiversity, whether computed for positive emotions, negative emotions or all emotions, were associated with better mental health, in the form of lower levels of self-reported depression symptoms, as well as better physical health, for example, levels of attendance at a doctor's (Quoidbach et al., 2014).

In related research, the tendency to use undifferentiated global emotion descriptors (particularly global negative descriptors) established from both self-report and ecological assessment data has been shown to be associated with a range of mental health disorders including Borderline Personality Disorder (Tomko et al., 2015), social anxiety (Kashdan & Farmer, 2014) and Major Depressive Disorder (Demiralp et al., 2012). Researchers have also recently found that greater diversity in specific positive emotion experience is associated with lower levels of systemic inflammation, providing a biological basis, perhaps, to help explain how positive emodiversity promotes physical health (Ong, Benson, Zautra, & Ram, 2017).

Complexity in an individual's experience of emotion, including emodiversity, is proposed to index the degree of complexity in the underlying conceptual structure of emotions. Theories of how such conceptual understanding might develop – for instance, the Levels of Emotional Awareness (LEA) approach (Lane & Schwartz, 1987) – posit that increased complexity of emotion experience emerges as a function of the underlying emotional schemas that have developed from early childhood onwards. In this analysis, differences across individuals in the complexity of their emotional lives reflect individual variation in the complexity of the underlying emotional representations that evolve throughout development (Lane & Nadel, 2000). This has led to the proposal (Werner-Seidler et al., 2018) that individuals who have suffered from chronic, sometimes lifelong, mental health problems may actually experience greater diversity of negative emotion experiences, due to their long history, familiarity and discourse with negative emotional constructs (Beck, Rush, Shaw, & Emery, 1979; Ratcliffe, 2015). In this view, negative emodiversity would reflect lifelong ‘expertise’ with negative emotions (Werner-Seidler et al., 2018) rather than some form of protection against mental health problems (cf. Quoidbach et al., 2014).

Werner-Seidler et al. (2018) investigated this proposal in individuals with chronic and lifelong depression. They found that, in contrast to those with elevated scores on depression measures in a general population sample (Quoidbach et al., 2014), chronically depressed individuals instead showed elevated levels of emodiversity for negative emotions relative to control participants who had never suffered depression. For positive emotions, emodiversity was reduced in the chronic, clinically depressed sample in line with the previous community findings. Werner-Seidler et al. (2018) proposed that, taken together, these findings suggest that, although negative emodiversity in the wider population may offer some protection against depression, for those with chronic clinical depression greater negative emodiversity will have emerged out of their long-term immersion in a complex, emotionally negative, personal narrative.

In the current study we sought to expand on these existing findings by examining the relationship between emodiversity and chronic and repeated trauma. Our rationale was first to establish whether the greater negative emodiversity associated with recurrent depression extended beyond this clinical syndrome. If, as Werner-Seidler et al. (2018) posit, greater negative emodiversity reflects lifelong ‘expertise’ with negative emotions, we would expect that the impact of severe trauma on emotional experience should produce a similar influence on emodiversity.

Second, recurrent depression is characterized by negative emotionality that infuses an individual's broadest representations of the self, the world and the future (Beck et al., 1979). Following trauma, the degree to which trauma-related material influences one's broader representations of the self, world, and future plays a key role in predicting the longer-term emotional state post-trauma, such that greater emotional disturbance is observed in those individuals for whom trauma-related appraisals, beliefs, and meaning permeates the broader autobiographical self. We were therefore interested in whether negative emodiversity in sexual trauma survivors would be potentially more constrained to discrete areas of trauma-related experience, or would in fact be characterized by elevated negative emodiversity across the entire autobiographical narrative, as has been found in recurrent depression (Werner-Seidler et al., 2018). For that reason, as in the prior clinical depression study (Werner-Seidler et al., 2018), we examined emodiversity within data that reflected the broadest notions of the self and the personal past.

Specifically, we examined emodiversity within two existing datasets. The first investigated the structure of self-concept (Clifford, Hitchcock, & Dalgleish, 2018a) and the second explored the organization of past autobiographical knowledge (Clifford, Hitchcock, & Dalgleish, 2018b) in female survivors of sexual abuse and assault, with chronic PTSD. In each study, we measured emodiversity for positive and negative emotion constructs in the trauma-exposed samples, relative to healthy control participants with no history of sexual trauma or PTSD.

Based on the earlier clinical depression study (Werner-Seidler et al., 2018), we hypothesised that individuals with a sexual trauma history would show elevated negative emodiversity and reduced positive diversity across both the structure of the self-concept and the structure of the life narrative.

## 2 Study 1: emodiversity within the self-structure in PTSD

### 2.1 Method

#### 2.1.1 Participants

Participants for Study 1 were drawn from the study by Clifford et al. (2018a) on the self-structure in sexual trauma survivors with PTSD (*n* = 23). Full details for inclusion and exclusion are reported in the original article.^2^ Briefly, fifteen of these participants were recruited from the Haven; A Sexual Assault Referral Centre (SARC) in London. They were invited to take part following attendance at the Haven follow-up clinic or during an assessment for counseling or psychological therapy. Eight participants were recruited from the MRC Cognition and Brain Sciences Unit Clinical Volunteer Panel – a database of approximately 400 community volunteers with a history of significant mental health problems. Volunteers are recruited to the panel via advertisements in local newspapers and through local clinics.

The participants with no history of PTSD and no reported history of sexual assault or abuse. (the Control Group; *n* = 22), were recruited from the MRC Cognition and Brain Sciences Unit Volunteer Panel. The Panel is a database of approximately 2000 community volunteers who have agreed to help with psychological research. Volunteers are recruited to the panel via advertisements in local newspapers.

PTSD diagnosis and history, and other Axis I and II psychiatric comorbidity according to the DSM-IV were determined by having participants complete the Structured Clinical Interview for the *DSM-IV* Axis I Disorders – Clinician Version (SCID, Version 2.0; First, Spitzer, Williams & Gibbon, 1996) and the Structured Clinical Interview for DSM-IV-TR Axis II Personality Disorders (Borderline, Avoidant and Dependant). To be eligible for the study, participants had to be fluent in English and over 18 years of age. Exclusion criteria comprised a diagnosis of substance dependence, a history of psychosis, and organic brain injury. No participants were excluded on these bases.

#### 2.1.2 Materials and measures

##### 2.1.2.1 Self-Structure Task

The Self-Structure Task is described in Clifford et al. (2018a). The task was adapted from Showers (1992; Showers & Kevlyn, 1999; Showers & Kling, 1996). Participants are given a description of how ‘self-aspects’ are defined (i.e., that our perception of our self can vary across different aspects of our lives, such as ‘self at work’, ‘self with friends’, ‘self as lover’, ‘self as mother’) and asked to identify and describe each of their different ‘self-aspects’. Participants are then given a deck of 48 cards, each containing a positive or negative trait adjective or phrase and asked to choose all cards which they felt were relevant in describing each of the self-aspects they had identified. Participants were instructed to use as many or as few adjectives as were relevant, and that repetitions were permitted. Four self-structure metrics (proportion of negative cards used, compartmentalization, and positive and negative redundancy) were derived and these metrics are reported in Clifford et al. (2018a).

##### 2.1.2.2 Emodiversity

Emodiversity across the card-sort data was calculated as in Werner-Seidler et al. (2018), using the formula below. This procedure was based on the formula provided by Quoidbach et al. (2014), which was originally derived from the Shannon biodiversity index (Shannon, 1948):

$$\text{Emodiversity}=\overset{s}{\underset{i=1}{\sum }}\left(p_{i}\;\text{x}\;\text{ln}\;p_{i}\right)$$

Where *s* = the total number of emotional trait adjective cards used in the card sort task by a given participant,^3^ and *p*_i_ is the proportion of *s* made up by each individual trait card. A value is calculated for each distinct trait card used, which is then imputed into the above formula. This formula takes into account both the number of traits reported as experienced (richness), as well as the degree to which different traits make up an individual's emotion experience (evenness/abundance). High values represent more diverse emotion experience. Emodiversity indices are calculated separately for negative and positive emotional traits.

#### 2.1.3 Procedure

Participants completed the tasks and measures individually and face-to-face with the experimenter, in a quiet testing room. Once they had consented, participants completed the SCID and then the Beck Depression Inventory (BDI-I; Beck, Ward, Mendelson, Mock, & Erbaugh, 1961) to assess current symptoms of depression, and the Centrality of Events Scale (CES -Negative; Berntsen & Rubin, 2006, 2007) to measure the extent to which a traumatic memory forms a central component of personal identity. Control participants anchored their responses on the CES to their most stressful life experience as a validity check that other stressful life events not included in the DSM definition of trauma had not adversely impacted the individual. The questionnaire measures were designed to validate our categorical participant group assignments by revealing significantly worse self-reported mood and symptoms of PTSD in the Trauma group relative to the Control Group. In a separate session, approximately a week later, participants completed the card-sorting task.

## 3 Results

### 3.1 Participant characteristics

As described in Clifford et al. (2018a), according to the SCID, all participants in the Trauma Group met criteria for chronic PTSD. Nine participants also met criteria for a diagnosis of Major Depressive Disorder (MDD), with a current Major Depressive Episode, 19 met criteria for a past Major Depressive Episode, one for current Panic Disorder (secondary to PTSD), four for current Borderline Personality Disorder (BPD) and two for current Avoidant Personality Disorder. In the Control Group, one participant met criteria for current MDD and five met criteria for a past Major Depressive Episode. The participant in the Control Group who met criteria for current MDD was excluded from analyses.

The remaining descriptive group data are presented in Table 1. Effect sizes are presented as Cohen's *d*s. All confidence intervals (CIs) are 95% confidence intervals. The Trauma and Control Groups did not significantly differ in age, *t*(42) = 1.41, *p* = .17, *d* = 0.43 [95% CI: −0.20, 1.06] or education level, *t*(42) = 1.75, *p* = .09, *d* = 0.53 [-0.10, 1.16]. There were the expected differences in BDI and CES scores between the groups, BDI: *t*(24.01) = 7.35, *p* < .001, *d* = 2.21 [1.42, 3.00]; CES: *t*(41) = 6.83, *p* < .001, *d* = 2.06 [1.29, 2.83].

**Table 1:** Means (and standard deviations) of participant characteristics in Study 1.

Category	Trauma Group (*n* = 23)	Control Group (*n* = 21^a^)
Years in education	14.87 (2.32)	16.05 (2.13)
Age (in years)	35.87 (14.03)	30.19 (12.54)
Beck Depression Inventory	24.77 (12.83)	3.95 (3.37)
Centrality of Events Scale	80.00 (15.49)	42.76 (20.07)

Note.

a data for one Control Group participant were set aside due to the presence of current MDD.

### 3.2 Emodiversity

**Fig. 1:**
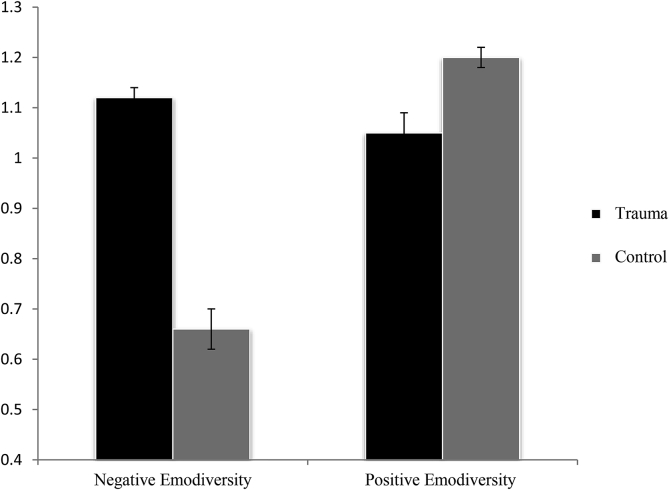
Mean (+1 SE) performance (y-axis) for the Trauma and Control Groups for Negative and Positive Emodiversity within the Self-Structure Task in Study 1.

The Positive and Negative Emodiversity findings are presented in Fig. 1. Across all participants, Negative and Positive Emodiversity scores were not significantly correlated, *r*(42) *=* .05, *p =* .76, demonstrating that it is valid to examine diversity indices separately across different valence domains (Quoidbach et al., 2014).

To examine patterns of Positive and Negative Emodiversity across groups, we conducted a mixed ANOVA with Valence (negative, positive) as the within-subjects factor, Group (Trauma, Control) as the between-groups factor, and Emodiversity index as the dependent variable (cf. Werner-Seidler et al., 2018). There was a significant main effect of Valence, *F*(1,42) = 26.63, *p <* .001, *d* = 1.56 [0.85, 2.27], such that there was greater Positive Emodiversity than Negative Emodiversity, and a significant main effect of Group, *F*(1,42) = 6.18, *p <* .02, *d* = 0.75 [0.11, 1.39], with greater Emodiversity in the Trauma Group relative to the Control Group. There was also a significant Valence × Group interaction, *F*(1,42) = 42.86, *p <* .001, *d* = 1.98 [1.22, 2.74]. In line with our predictions, follow-up ANOVAs demonstrated significantly greater Negative Emodiversity for the Trauma group, *F*(1, 42) = 27.03, *p* < .001, *d* = 1.57 [0.86, 2.28], and significantly greater Positive Emodiversity for the Control Group, *F*(1, 42) = 4.48, *p* = .04, *d* = 0.63 [-0.01, 1.26].

### 3.3 Emodiversity and emotional negativity/positivity within the Self Structure

We next sought to evaluate whether these differential emodiversity effects across groups were a function of greater overall endorsement of emotional words (cf. Quoidbach et al., 2014; Werner-Seidler et al., 2018). We first looked at Negative Emodiversity. As a function of our formula to calculate emodiversity, higher levels of Negative Emodiversity are linked to the selection of a greater number of negative cards overall in the card-sort, so we cannot simply use the latter when trying to disentangle emodiversity and general negativity. However, we can compute the number of times a diverse set of selected negative cards is used *repeatedly* by each participant across their Self Structure (i.e., Negative Repetitions). To calculate this, we used the following formula: Negative Repetitions = (Total number of negative cards used – number of distinct negative cards used)/number of distinct negative cards used.^4^ The Trauma Group had a higher mean level of Negative Repetitions (*M* = 0.94, *SD* = 0.85) relative to the Control Group (*M* = 0.47, *SD* = 0.40), *t*(32.11) = 2.38, *p* = .02; *d* = 0.72 [0.08, 1.36], reflecting a greater repeated endorsement of their diverse set of negative cards across their self-aspects.

We next entered these Negative Repetition scores as a covariate into our main analyses to see if the greater repeated negativity of the Trauma group mitigates our core findings. The pattern of core emodiversity results was unchanged, with a significant Valence × Group interaction, *F*(1,40) = 34.65, *p <* .001, *d* = 1.78 [1.04, 2.51] and follow-up comparisons showing greater Negative Emodiversity for the Trauma Group compared to the Control Group, *F*(1,40) = 18.37, *p* < .001, *d* = 1.29 [0.61, 1.97], but lower levels of Positive Emodiversity, *F*(1,40) = 8.05, *p* = .007; *d* = 0.85 [0.21, 1.49].

Using the same formula, we computed the number of times a diverse set of selected positive cards was used repeatedly by each participant across their self-structure (i.e., Positive Repetitions). Interestingly, the Trauma Group actually had a marginally higher mean level of Positive Repetitions reflecting a greater repeated endorsement of their diverse set of positive cards across their self-aspects (*M* = 1.33, *SD* = 1.37) relative to the Control Group (*M* = 1.25, *SD* = 0.81), although this difference was not significant *t*(42) = 0.22, *p* = .83, *d* = 0.07 [-0.55, 0.69].

We then entered these Positive Repetition scores as a covariate into our core analyses and again the pattern of results was unchanged, with a significant Valence × Group interaction, *F*(1,41) = 43.21, *p* < .001, *d* = 1.98 [1.22, 2.74], and follow-up comparisons showing significantly greater Negative Emodiversity for the Trauma Group, *F*(1, 41) = 27.35, *p* < .001, *d* = 1.58 [0.87, 2.29], and significantly greater Positive Emodiversity for the Control Group, *F*(1, 41) = 6.56, *p* = .01, *d* = 0.77 [0.13, 1.41].^5^

## 4 Discussion

Study 1 investigated the diversity of endorsed emotion descriptors – emodiversity – across the self-structure in individuals with chronic PTSD following sexual trauma relative to healthy controls with no history of such experiences. In line with our predictions, we found that, relative to healthy controls, the sexual trauma survivors endorsed a greater diversity of emotion descriptors within the negative affective domain and a reduced diversity of positive descriptors. These effects appeared to be independent of the frequency of endorsement of those descriptors. These findings mirror the emodiversity results in individuals with a primary diagnosis of chronic Major Depressive Disorder reflecting on their autobiographical past (Werner-Seidler et al., 2018). Thus, findings suggest that elevated negative emodiversity may be a broader marker of emotional disturbance, rather than a specific feature of major depression.

The current findings and the previous findings in individuals with depression (Werner-Seidler et al., 2018) are discrepant to earlier results with community samples of mostly healthy individuals. Quoidbach et al. (2014) had shown an association between greater negative emodiversity and reduced symptoms of depression, with the authors arguing that emodiversity thereby serves as a protective factor against mental health problems. The present results suggest that, in chronic clinical samples, different processing dynamics might be operating with greater negative emodiversity in these groups reflecting immersed ‘expertise’ with negative affective experiences.

In Study 2 we sought to replicate the present results using the same task as the prior findings in groups with clinical depression (Werner-Seidler et al., 2018). In the task, participants endorse a diversity of emotion descriptors with reference to the entirety of their past autobiography as opposed to their current self-concept.

## 5 Study 2: emodiversity within the life structure in sexual trauma survivors

### 5.1 Method

#### 5.1.1 Participants and procedure

Participants in Study 2 were drawn from Clifford et al. (2018b) and full details on participant recruitment and inclusion criteria are provided there.^6^ These criteria are identical to those for Study 1. The Trauma and Control groups in Clifford et al. (2018b) were not matched for years in education. Due to the likely importance of education as an influence on conceptual measures such as emodiversity, prior to completing analyses we excluded data from four participants from the original Trauma Group in Clifford et al. (2018b) who had a low number of years in education and one member of the Control Group with a high number of years in education, to ensure a better balance across groups.

This gave us a Trauma Group of *n* = 23 and a Control Group of *n* = 22. Of this Trauma Group, 15 participants were recruited from the Haven; and eight were recruited from the MRC Cognition and Brain Sciences Unit Clinical Volunteer Panel.

Nine of the participants in the Trauma Group and 8 participants in the Control Group were also participants in Study 1. The pattern of results remained the same when these participants were excluded from the analyses.

The procedure was as for Study 1 except for the Life Structure Task being administered in place of the Self Structure Task.

#### 5.1.2 Life Structure Task

The Life Structure Task is described in detail in Clifford et al. (2018b). In brief, participants were asked to think back over their life and divide it into chapters. Participants then provided a relevant heading for each chapter and completed the card sort task as outlined in Study 1, this time allocating cards to life chapters instead of self-aspects. Positive and Negative Emodiversity were calculated as described in Study 1.

## 6 Results

### 6.1 Descriptive group data

According to the SCID, in the Trauma Group, all participants met criteria for PTSD, five participants also met criteria for current MDD, 22 met the criteria for a past Major Depressive Episode, seven for current Panic Disorder (secondary to PTSD), two for current Agoraphobia, five for current Borderline Personality Disorder, and two for current Avoidant Personality Disorder. In the Control Group, three participants met criteria for a *past* episode of MDD and one for current panic disorder.

The descriptive group data are presented in Table 2. The groups did not differ in age, *t*(43) = 0.27, *p* = .79, *d* = 0.08 [-0.53, 0.69], nor years in education, *t*(43) = 1.90, *p* = .07, *d* = 0.58 [-0.04, 1.20]. There were the expected differences in BDI and CES scores between the Trauma and Control groups – BDI: *t*(22.89) = 8.79, *p* < .001, *d* = 3.67 [2.65, 4.68]; CES: *t*(42.10) = 8.81, *p* < .001, *d* = 2.72 [1.86, 3.58].

**Table 2:** Means (and standard deviations) of participant characteristics in Study 2.

Category	Trauma Group (*n* = 23)	Control Group (*n* = 22)
Years in Education	14.91 (2.83)	16.36 (2.26)
Age (in years)	36.70 (13.48)	35.55 (15.05)
Beck Depression Inventory	25.09 (12.69)	1.59 (1.76)
Centrality of Events Scale	82.09 (16.43)	36.55 (18.17)

### 6.2 Emodiversity

**Fig. 2:**
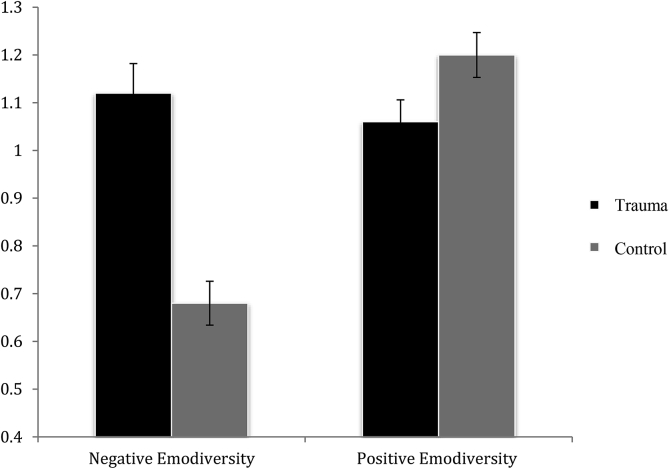
Mean (+1 *SE*) performance (*y*-axis) for the Trauma and Control Groups for Negative Emodiversity and Positive Emodiversity across the Life Structure in Study 2.

The emodiversity data for the Life Structure Task are presented in Fig. 2. As in Study 1, across all participants, Negative and Positive Emodiversity scores were not significantly correlated, *r*(45) *=* -.05, *p =* .76.

As for Study 1, to examine patterns of Positive and Negative Emodiversity across groups, we conducted a mixed ANOVA with Valence (negative, positive) as the within-subjects factor, Group (Trauma, Control) as the between-subjects factor, and the emodiversity index as the dependent variable. As in Study 1, there was a main effect of Valence, *F*(1,43) = 9.39, *p =* .004, *d* = 0.91 [0.27, 1.55], with significantly higher Positive Emodiversity across the Life Structure. However, unlike Study 1, there was no significant main effect of Group, *F* < 1. As hypothesised and in line with the results of Study 1, there was a significant Valence × Group interaction, *F*(1,43) = 11.98, *p <* .001, *d* = 1.03 [0.38, 1.68]. Follow-up ANOVAs again showed significantly greater Negative Emodiversity in the Trauma Group, *F*(1, 43) = 5.42, *p* = .03, *d* = 0.69 [0.06, 1.32], and significantly greater Positive Emodiversity in the Control Group, *F*(1, 43) = 5.60, *p* = .02, *d* = 0.71 [0.08, 1.34].

### 6.3 Emodiversity and negativity/positivity

As for Study 1, we repeated our main analyses covarying Negative and Positive Repetitions. The Trauma Group (*M* = 1.29, *SD* = 0.91) had a numerically higher mean level of Negative Repetitions relative to the Control Group (*M* = 1.25, *SD* = 1.27), but this difference was not significant *t*(43) = 0.19, *p* = .91, *d* = 0.06 [-0.55, 0.67]. Covarying for Negative Repetition scores in our main emodiversity analyses again left the pattern of results unchanged, with a significant Valence × Group interaction, *F*(1,42) = 12.02, *p* = .001 *d* = 1.03 [0.38, 1.68] and follow-up paired comparisons showing greater Negative Emodiversity, *F*(1,42) = 5.61, *p* = .02; *d* = 0.71 [0.08, 1.34], but lower levels of Positive Emodiversity, (1,42) = 5.66, *p* = .02; *d* = 0.71 [0.08, 1.34], in the Trauma Group compared to the Control Group.

The Control Group (*M* = 2.52, *SD* = 1.43) had a significantly higher mean level of Positive Repetitions relative to the Trauma Group (*M* = 1.46, *SD* = 1.57), *t*(42.91) = 2.37, *p* = .02; *d* = 0.72 [0.09, 1.35]. Entering these Positive Repetition scores as a covariate into our main analyses did impact the pattern of significant results. There remained a significant Valence × Group interaction, *F*(1,42) = 8.87, *p* = .005; *d* = 0.89 [025, 1.53], and the follow-up paired comparison continued to show greater Negative Emodiversity, *F*(1,42) = 5.94, *p* = .02; *d* = 0.73 [0.10, 1.36], for the Trauma Group relative to the Control Group. However, there was no longer a significant group difference for Positive Emodiversity, *F*(1,42) = 1.91, *p* = .17, *d* = 0.41 [-0.21, 1.03].^7^

## 7 Discussion

The results of Study 2 replicated the critical negative emodiversity results from Study 1, demonstrating that our participants with chronic PTSD following sexual trauma exhibited greater negative emodiversity relative to healthy controls with no such history. However, once we adjusted analyses to account for the frequency with which the diverse emotion descriptors were employed, the group difference in positive emodiversity was no longer significant.

These results suggest that within a different autobiographical domain (i.e., the life narrative relative to self-concept), chronic PTSD following sexual trauma remains characterized by greater negative emodiversity, independent from the frequency with which negative emotion descriptors are endorsed. In the positive domain, the findings support the notion that positive emodiversity is also reduced in our sample with a sexual trauma history but that such reduced positive emodiversity may go hand-in-hand with a reduced endorsement of positive emotion terms more generally.

## 8 General discussion

Across two studies we showed that sexual trauma survivors with a diagnosis of chronic PTSD endorsed a greater diversity of negative emotion descriptors when describing either their current self-structure (Study 1) or their autobiographical past (Study 2), relative to healthy control participants who had no such trauma history. This greater negative emodiversity appears to be independent of the *frequency* of endorsement of negative descriptors. Relatedly, in both studies we showed that sexual trauma survivors also exhibited reduced positive emodiversity relative to controls. In Study 1, this appeared to be independent of the frequency of endorsement of those positive descriptors. However, when examining the life-structure in Study 2, the group difference was no longer significant when adjusting for endorsement frequency, suggesting that the frequency and diversity of positive emotion descriptor use are highly associated.

The current results mirror those in another chronic mental health disturbance – primary recurrent Major Depressive Disorder (Werner-Seidler et al., 2018) – using the same life structure methodology as in the current Study 2. This suggests that valence-based differences in emodiversity are not constrained to a primary presentation of depression, but may also be related to mental health distrunbances in the aftermath of severe trauma. The current results, along with those from Werner-Seidler et al. (2018), are somewhat divergent from earlier studies using community samples (Quoidbach et al., 2014) which suggested that greater emodiversity (including in the negative valence domain) is associated with fewer symptoms of mental ill health.

As discussed in the Introduction, taken together these studies suggest that in a wider community context, greater negative emodiversity may be associated with protection against mental health difficulties, but that this protective factor is not evident for chronically unwell individuals, including those exposed to severe trauma. Once significant difficulties become established and consolidated over time, the chronic immersion in negative affective self-referent material and the relative paucity of positive self-reference material may see that those exposed to severe sexual trauma and/or a chronic emotional disorders such as PTSD and recurrent MDD develop ‘expertise’ in negative affective experiences. This would afford concomitantly greater diversity in the ways that such negative experiences are described and articulated.

It is proposed that greater emodiversity in community samples (Quoidbach et al., 2014) may lead to better mental health as more affective differentiation in the description of emotional life reflects more granularity in the emotional experiences themselves. This is in turn associated with an enhanced ability to target emotion regulation strategies at specific emotion experiences, with consequent enhancement of overall emotion regulation and of mental health (e.g. Feldman-Barrett et al., 2001; Feldman-Barrett & Gross, 2001). The current data do not necessarily contradict this analysis. It remains possible that chronic emotional disturbance is in fact associated with an enhanced *underlying capacity* for emotion regulation but that emotions in the day-to-day remain more dysreguylated as a simple function of the severity and dysfunctionality that unwell individuals are trying to regulate. In a sense this would not be surprising as trauma survivors with PTSD report spending large amounts of their time trying to regulate aversive negative cognitions and affect. They are therefore highly practiced with the techniques even if they are often experienced as ineffectual. Although this view has not been investigated directly (most studies examine self-reported emotion regualtion using questionnaires which confounds capacity and day-to-day experience; e.g. Ehring & Quack, 2010) there is some support in the literature. For example, young adults who have been exposed to childhood adversity perform better, and show reduced neural engagement of emotion regulatory circuitry in the brain, on a laboratory emotion regualtion task relative to participants who report no such adversity (Schweizer et al., 2015; see also Sheperd &Wild, 2014).

The studies reported here, either together or individually, have some possible limitations that merit discussion. We did not ask participants to generate their own descriptive words for the cards used in the self- and life-structure tasks. This was to ensure that there were equal quantities of positive and negative descriptors matched for emotional intensity, word length and frequency. Future studies could adopt an ideographic approach, potentially in combination with experience sampling methods, to provide a richer personalized evaluation of diversity. Most notably, in both studies our sexual trauma survivors all experienced chronic PTSD, and selecting such a severe trauma placed constraints on our selection of control participants. It is prohibitively difficult to find survivors of sexual trauma with only a few or no significant symptoms of past or current PTSD to serve as a trauma-matched control sample. We therefore had to recruit a comparison sample who both had no history of sexual trauma *and* no history of PTSD to any trauma. This precludes disentangling whether it is the presence of PTSD rather than the trauma history itself which accounts for our results. Similarly, although Werner-Seidler et al.’s (2018) depressed participants had not experienced repeated sexual trauma, some had experienced traumatic events. A direct comparison against a depressed group with no trauma history would therefore allow firmer conclusions from our results, and allow further disentanglement of the relative effects of depression, sexual trauma, and PTSD.

A limitation of the life structure task is that, although it focuses on the whole life narrative, it remains retrospective. Similarly, while the self-structure task lacks this historical element, it does require participants to reflect on self-aspects that they may not currently have immediate cognitive access to. It is therefore possible that a methodology that permitted contemporaneous consideration of past life chapters or of alternative current self-aspects may have generated a different set of results.

Finally, the sample sizes for each study were modest, as is often the case for hard-to-recruit clinical samples. However, the replication of the results across two different versions of the card sorting task mitigates these sampling concerns. The study samples were also all female. It would therefore be important to replicate the current findings with larger samples including individuals with PTSD who have experienced different traumas, and who are male or non-binary.

In summary, across two studies we showed that PTSD following sexual trauma is associated with the endorsement of a greater diversity of negative emotion descriptors and a reduced diversity of positive emotion descriptors when describing either the self or the autobiographical past relative to healthy control participants with no history of sexual trauma. These results, along with similar findings in individuals with chronic depression (Werner-Seidler et al., 2018), suggest that elevated negative emodiversity could potentially be a transdiagnostic marker of chronic emotional disturbance. This contrasts somewhat with proposals in the emotion literature that enhanced negative emodiversity represents a protective factor against mental health difficulties.

## Author note

This work was funded by the UK Medical Research Council (MRC; grant number SUAG/006/RG91365) and supported by the National Institute for Health Research Cambridge Biomedical Research Centre.

## Footnotes

2 As we made use of existing data, we were unable to complete an a priori power calculation. However, posthoc power analysis using the effect size obtained for our hypothesised effect indicated that we achieved 100% power (α = 0.05, two-tailed).

3 This is the total number of adjectives used, such that if a trait-card is used twice, it is counted twice. For example, if the individual used 4 cards 5 times (20 endorsements), and one card 3 times (3 endorsements), *s* = 23.

4 Data for one control participant were set aside as they endorsed zero negative cards.

5 Because the groups trended towards being significantly different in terms of education history, we repeated these analyses covarying years in education and the results were unchanged, *F*s > 5, *p*s < .03.

6 As we made use of existing data, we were unable to complete an a priori power calculation. However, posthoc power analysis using the effect size obtained for our hypothesised effect indicated that we achieved 92% power (α = 0.05, two-tailed).

7 As in Study 1, because the groups trended towards a different in education history, we repeated the above analyses this time with years in education as a covariate. The results were comparable: Negative Emodiversity covarying negative repetitions and years in education, *F* (1,41) = 3.73, *p* = .06; Negative Emodiversity covarying positive repetitions and years in education, *F* (1,41) = 4.69, *p* = .04; Positive Emodiversity covarying negative repetitions and years in education, *F* (1,41) = 3.87, *p* = .05; and Positive Emodiversity covarying positive repetitions and years in education, F (1,41) = 1.27, *p* = .27.
